# The mediating influence of perceived workplace ostracism on the relationship between interpersonal distrust and knowledge hiding and the moderating role of person-organization unfit

**DOI:** 10.1016/j.heliyon.2023.e20008

**Published:** 2023-09-09

**Authors:** Fatima Saeed Al-Dhuhouri, Faridahwati Mohd Shamsudin

**Affiliations:** College of Business Administration, University of Sharjah, Sharjah City, United Arab Emirates

**Keywords:** Interpersonal distrust, Perceived workplace ostracism, Knowledge hiding, Person-organization unfit

## Abstract

Experiencing ostracism at work is traumatic, adversely impacting employees' mental health, attitudes, and behaviors. Although the effects of workplace ostracism have captured academic interest, holistic models identifying its antecedents' determinants and ramifications are scarce. This research conducts a holistic investigation of perceived workplace ostracism by evaluating how interpersonal distrust influences it and the knock-on effect on knowledge hiding. Moreover, it investigates the moderating role of person-organization unfit in the proposed perceived workplace ostracism–knowledge hiding relationship. Data gathered from 242 employees in the United Arab Emirates was analyzed via partial least squares structural equation modeling (PLS-SEM). The findings reveal that interpersonal distrust positively affects perceived workplace ostracism, which in turn drives knowledge hiding. Additionally, person-organization unfit moderates perceived workplace ostracism's influence on knowledge hiding, with high unfit exacerbating the effect. We discuss our findings' practical and theoretical implications and suggest future avenues for research.

## 1 Introduction

Perceived workplace ostracism refers to an employee feeling excluded by colleagues during social interactions or workplace activities that should involve them [59,77,78]. It includes situations when their colleagues avoid them, refuse to talk to them, or even exclude them from conversations that demand participation [26]. Various studies suggest that workers frequently encounter workplace ostracism. According to Fox and Stallworth [27], 66% of full-time employees surveyed over five years reported having been ignored, while 29% said they had experienced colleagues exiting the room when they entered. In O'Reilly et al.’s [54] survey of 1300 working-age Americans, 71% had experienced social exclusion in the preceding six months. Thus, we may surmise that workplace ostracism is prevalent [44].

Workplace ostracism is a form of mistreatment that manifests as social rejection [37], limiting chances for social contact and preventing employees from establishing trusting, long-lasting connections within the organization [39]. Such adverse experiences are baleful as they thwart employees' belongingness, control, meaningful existence, and self-esteem needs, consequently endangering their well-being, attitude, and behavior [79]. This phenomenon's adverse consequences have triggered substantial scholarly interest [37,48].

We must understand workplace ostracism's antecedents to address and curb its adverse effects. Past studies tended to investigate its causes or effects separately [37], yet effective interventions demand a holistic understanding of this phenomenon. Considering this gap, we offer a comprehensive model that investigates both factors in a single study. Our primary contribution lies in our holistic model that incorporates perceived workplace ostracism as a mediator to analyze the antecedents and consequences, thereby informing our theoretical understanding of ostracism's causes and impacts on organizations.

Drawing on social identity theory (SIT) [70], which argues that how individuals perceive and identify with others informs their attitudes and behaviors, we examine interpersonal distrust as a potential antecedent for perceived workplace ostracism. We focus on interpersonal distrust because workplace ostracism is embedded in social relationships, and distrust disrupts the core of interpersonal workplace interactions [31]. We hereby respond to the call to investigate interpersonal distrust, which has featured less prominently in organizational research than interpersonal trust [51]. We postulate that employees who distrust colleagues often exit the prototypical (trustful) group, making them more likely to dissociate and identify as outgroup members. This experience triggers feelings of ostracism, producing a negative response. The negative reciprocity principle [30] implies that ostracized workers perform negative behaviors because individuals reciprocate in kind in social relationships to restore balance [18]. Prior literature on workplace ostracism highlights the adverse consequences of this phenomenon, including reduced performance [25], escalated interpersonal deviance [1], and decreased voice behavior [80]. This study seeks to contribute to this research by examining knowledge hiding as a behavioral consequence of perceived ostracism. An organization's long-term viability critically depends on its capacity to manage knowledge [22,62], with studies showing improved organizational performance when employees consistently share knowledge [4].

We test our model further by incorporating a boundary condition to help understand *when* perceived ostracism's impact on employees' knowledge hiding is stronger. Feeling like outgroup members, employees with perceived ostracism may hide knowledge because they believe that their values are incongruent with the organization's [45]. Alignment is crucial for effective organizations [19], yet whether person-organization unfit further exacerbates ostracized employees' propensity to hide knowledge is unknown.

### 1.1 Objectives and significance of the study

**Fig. 1:**
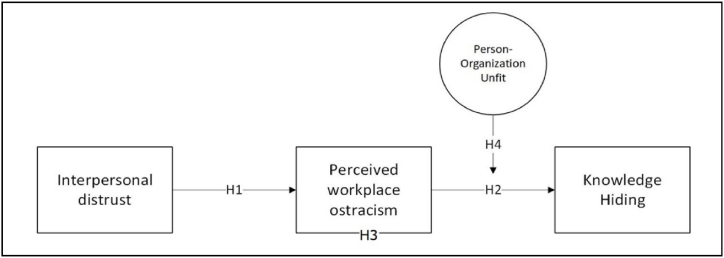
The theoretical model of the study.

This study proposes a holistic model of the causes and ramifications of perceived workplace ostracism (see Fig. 1), systematically exploring (a) interpersonal distrust's influence on perceived workplace ostracism, (b) perceived workplace ostracism's impact on employees' knowledge hiding, (c) perceived workplace ostracism's mediating role in the interpersonal distrust–knowledge hiding relationship, and (d) person-organization unfit's moderating role in the perceived workplace ostracism–knowledge hiding relationship.

This study contributes to the burgeoning ostracism literature by developing and evaluating a holistic model that incorporates workplace ostracism as a mediator to analyze its antecedents and consequences, drawing on SIT [70] and the negative reciprocity principle [30]. Secondly, considering the research gap on the drivers of knowledge hiding, we investigate *how* and *why* interpersonal distrust and perceived workplace ostracism incite this behavior; additionally, we examine if and how the boundary condition of person-organization unfit heightens the effect of perceived workplace ostracism on knowledge hiding.

Finally, we explore how perceived workplace ostracism influences knowledge hiding in the United Arab Emirates (UAE). The UAE has a diverse workforce, as its population is primarily composed of expatriates from over 200 nationalities [28]. Knowledge-hiding behavior in diversified workplaces is deleterious because it conceals diversified and rich knowledge and expertise, potentially hindering organizational success. Furthermore, the country seeks to leverage knowledge to drive its knowledge-based economy [52]. Thus, this study is crucial to meeting this aspiration.

## 2 Literature review and hypotheses development

### 2.1 Perceived workplace ostracism

Perceived workplace ostracism is an employee's subjective belief that they are the target of negative behavior from one or more organization members, indicating a disparity between the employee's desired and actual levels of social engagement [26]. Ostracism is pervasive in society, including the workplace [83]. It reduces opportunities for interpersonal interactions and hampers long-term and meaningful employee relationships [39]. The workplace victimization relational model by Aquino and Lamertz [3] reveals a two-way interaction between the victim in the submissive or provocative role and the offender in the dominant or reactive role; organizational factors such as culture, power differences, and social capital are also influential [3]. Prior research has focused on the consequences of workplace ostracism from the victims' perspective due to its effects on mental and physical health [37]. Our study similarly adopts the victim's perspective in holistically examining the phenomenon.

While scarce research investigates workplace ostracism's causes, the victim's attributes and environmental aspects are established systematic predictors [37]. Five significant personality traits are the most researched individual predictors of workplace ostracism [37], although interpersonal distrust, a critical social factor [60,69], has received little attention beyond its positive effect on workplace ostracism [42,85]. Building on SIT [70], we enrich the current literature by examining interpersonal distrust as an antecedent of perceived ostracism.

Workplace ostracism has various personal, relational, and organizational outcomes [66]. On the personal level, it causes burnout [57] and deteriorates psychological health [65]. At the relational level, it lowers family satisfaction [47] and thwarts knowledge-sharing behavior [71], obstructing organizational performance [43].

### 2.2 Interpersonal distrust and perceived workplace ostracism

Trust is a person's tendency to expose themselves to harm from another, believing that the latter will act beneficially even though they have little influence over them [49]. Interpersonal trust significantly affects human conduct and interactions [60,69] and is highly influential for group performance [29,87]; in the workplace, it can boost group members' collaboration capacity [23,29,88]. Meanwhile, interpersonal distrust is a person's belief that another might harm them due to a lack of concern about their welfare, plans to undertake harmful actions, or simple hostility [31]. Interpersonal distrust disrupts group progress because distrusting team members is likely to divert their energy toward monitoring colleagues' behavior due to the perceived threat to cooperative behavior [23].

To clarify how interpersonal distrust influences perceived workplace ostracism, we draw on social identity theory [70], which explains the cognitive processes underpinning individuals' identification with and behavior within social groups. It argues that individuals’ attitudes and behaviors are influenced by how they perceive and identify with others. Building on this, we propose that employees who distrust their colleagues will likely exit the prototypical (trustful) group, causing them to dissociate and identify as outgroup members, resulting in perceived workplace ostracism. Karim et al. [42] argue that when distrust pervades the workplace, employees will be less inclined to engage in relationships with untrustworthy colleagues, leading to reduced participation in collaborative exchanges and increasing feelings of ostracism. Recent findings suggest interpersonal distrust positively correlates with perceived workplace ostracism [64,85]. Thus, we postulate the following:

Hypothesis 1 Interpersonal distrust is positively associated with perceived workplace ostracism.

### 2.3 Perceived workplace ostracism and knowledge hiding

Knowledge sharing is a cornerstone of corporate success [58]. Effective information dissemination not only contributes to organizational growth but also crucially determines the organization's ability to compete [2,24,52]. Knowledge hiding, i.e., an employee's deliberate attempt to withhold or hide requested or valuable information [21], is pervasive throughout organizations [52]. While the sharing and hiding of knowledge were formerly thought to be two extremes of one continuum, scholars have recently argued that they are distinct concepts [9,21,52].

Understanding the distinction between knowledge hiding, knowledge hoarding, and counterproductive workplace behaviors is crucial. Knowledge hiding denotes the deliberate withholding of information requested by another party, distinguishing it from knowledge hoarding, which is proactively amassing information that another party might request [52,75]. Meanwhile, Spector and Fox [67] define counterproductive behaviors as voluntary behaviors committed by employees that aim to hurt companies and their stakeholders. Knowledge hiding does not always involve malicious intent, e.g., when employees use it to protect or to avoid hurting another's feelings [13,21].

The antecedents of knowledge hiding have received less research attention than knowledge hiding's detrimental effects [52,58,86]. Extant literature reveals that perceived workplace ostracism induces knowledge hiding [58,86], wherein employees' withholding of crucial information inhibits organizational development [21,58,86]. The reciprocity norms govern social relationships [30], wherein individuals are assumed to treat others in the same manner as they were treated. Specifically, individuals are expected not to harm those who have assisted them (positive reciprocity) but may retaliate against those who have wronged them (negative reciprocity) [30]. The perception of being a victim of ostracism is an unpleasant interpersonal experience, and employees may retaliate accordingly by inflicting harm, e.g., by hiding requested knowledge. Based on this, we hypothesize that:

Hypothesis 2 Perceived workplace ostracism is positively associated with knowledge hiding.

### 2.4 The mediation of perceived workplace ostracism

Organizational research investigates mediation effects as they can unravel mechanisms linking independent and dependent variables [81]. Here, we propose that perceived workplace ostracism mediates the interpersonal distrust–knowledge hiding relationship. Employees are prone to perceiving ostracism when interpersonal trust is scarce [42,64,85]. Building on SIT [70], distrustful employees exit the prototypical (trustful) group, leading them to identify as outgroup members. Further, building on the negative reciprocity principle [30], employees with perceptions of ostracism may retaliate by inflicting harm to restore their social functioning and system [18], potentially via knowledge hiding.

Hence, we propose that interpersonal distrust indirectly affects employee knowledge hiding behavior through perceived workplace ostracism. Building on the prior two hypotheses, we propose:

Hypothesis 3 Perceived workplace ostracism mediates the relationship between interpersonal distrust and knowledge hiding.

### 2.5 Person-organization unfit as a moderator

Employees' fitness with their organization influences their attitudes and behaviors. “Person-organization fit” is the match between the employee's and the organization's values [45]. Value compatibility facilitates open lines of communication because it establishes a common framework for defining, classifying, and interpreting situations [50,63], facilitating information transmission, and decreasing misunderstandings [41]. Shared norms also imply that employees use mutual cognitive processing methods, including encoding and decoding verbal and nonverbal cues for communication [76]. Thus, if employees perceive inadequate value compatibility (e.g., person-organization unfit), they will define, classify, and interpret events differently, complicating communication and increasing misunderstanding. It can intensify outgroup feelings, potentially reducing information sharing [15].

Person-organization unfit could exacerbate perceived workplace ostracism's influence on knowledge hiding. The socialization literature contends that individuals are likely to enter and maintain preferred relationships in an organization when their values align with the organization's [12], implying a feeling of belongingness and identification with the organization and its members, consistent with social identity theory [70]. Inversely, employees whose values do not align risk social isolation, reinforcing their outgroup feelings. When they lack belongingness, employees are likely to disobey organizational rules, e.g., by engaging in knowledge hiding [40]. We hypothesize that:

Hypothesis 4 Person-organization unfit moderates the positive association between perceived workplace ostracism and knowledge hiding, whereby the association is stronger when the unfit is higher.

Fig. 1 presents the hypothesized relationships.

## 3 Methodology

### 3.1 Participants and procedure

We collected survey data from employees across different sectors (private, semi-government, and government) in the UAE via convenience sampling. Using the professional and personal networks of one of the authors, a DBA student, this sampling technique ensured easy data access, geographical proximity, and availability. It has been employed in past studies [7,8,52,73].

We used paper-based questionnaires, initially designed in English, and subsequently translated into Arabic. To ensure translation accuracy and item comprehension consistency, the questionnaires were back-translated [10]. The participants were offered both the Arabic and English versions.

The questionnaire included a participant information sheet as a cover letter outlining the study objectives, voluntary participation, assurances of participant anonymity and privacy, and clarification that participation entailed no known risks. It was done to address Common Method Variance [56], a frequent issue in survey research [8].

The questionnaire consisted of two parts. The first captured the participants’ demographic data, encompassing age group, gender, highest educational attainment, sector of employment, and tenure. The second part used 20 items to probe the four study variables (interpersonal distrust, perceived workplace ostracism, knowledge hiding, and person-organization unfit).

Four hundred eight questionnaires were manually distributed to participants during site visits. After three weeks, 257 questionnaires were returned (63% response rate). After removing missing and inconsistent responses, 242 valid responses remained for data analysis, producing a 59% response rate.

Table 1 presents the respondents' demographic profiles. There were more males (66.9%) than females (33.1%). Regarding the age group, 33.1% were 18–30 years old, 44.2% were 31–40 years old, and 22.7% were older than 40. Regarding education level, all participants were educated: 38.9% had a college degree or below, 43.4% had a bachelor's degree, 14% had a master's degree, and 3.7% had a doctoral degree. Regarding tenure, most had a tenure of one to five years and 11–20 years, and few had more than 20 years (7.4%). Finally, the majority worked in the public sector (80.2%), while 11.6% and 8.3% worked in the semi-government and private sectors, respectively.

**Table 1:** Participants’ demographic profiles.

Items	N	%
***Gender***		
**Male**	162	66.9
**Female**	80	33.1
Total	**242**	**100**
***Age group***		
**18–30**	80	33.1
**31–40**	107	44.2
**Above 40**	55	22.7
Total	**242**	**100**
***Education level***		
**College degree or lower**	94	38.9
**Bachelor's degree**	105	43.4
**Master's degree**	34	14.0
**Doctoral degree**	9	3.7
Total	**242**	**100**
***Tenure***		
**1**–**5 years**	79	32.6
**6**–**10 years**	66	27.3
**11**–**20 years**	79	32.6
**Above 20 years**	18	7.4
Total	**242**	**100**
***Sector***		
**Public**	194	80.2
**Semi-government**	28	11.6
**Private**	20	8.3
Total	**242**	**100**

### 3.2 Measurements

Established instruments from the literature were used to measure the study's variables. Participants showed their level of agreement or disagreement with the items using a five-point Likert scale (‘1’ for ‘strongly disagree’ to ‘5’ for ‘strongly agree’) for all instruments. The measures and their psychometric properties are outlined in the following:

*Interpersonal distrust.* Three items derived from Baltatescu [5] measure this concept. An example was, “Most people cannot be trusted.”. This variable has a Cronbach's alpha of 0.72 [5].

*Perceived workplace ostracism*. This concept was measured with ten items derived from Ferris et al. [26]. A sample item was, “You noticed others would not look at you at work.”. This variable has a Cronbach's alpha of 0.92 [26].

*Knowledge hiding*. Peng's [55] four-item scale was employed to measure this concept. An example was, “I do not want to transfer personal knowledge and experience to others.”. This variable has a Cronbach's alpha of 0.89 [55].

*Person-organization unfit*. Cable and DeRue [14] devised a three-item scale to quantify this construct. An example was, “The things that I value in life are not similar to what my organization values.”. This variable has a Cronbach's alpha of 0.90 [14].

*Control variables***.** Because of their known effects on perceived workplace ostracism, gender, age range, tenure, and highest level of education were utilized as control variables [37].

### 3.3 Common Method Variance

Because the data were self-reported and cross-sectional, Common Method Variance was a concern. In addition to the abovementioned procedural remedy, we used statistical procedures, particularly the primary axis factoring method, to conduct Herman's single-factor test. The first factor explained 46% of the variance, indicating no significant threat of Common Method Variance.

## 4 Analysis and results

Instrument validation and hypothesis testing were conducted using partial least squares structural equation modeling (PLS-SEM) via Smart-PLS 3.0 Software. The analysis simultaneously calculated the dimensions and basic model by conducting confirmatory factor analysis and regression [33]. The instruments’ reliability and validity were assessed to ascertain the measurement model, and structural model analysis was used to test the hypotheses [35]. Smart-PLS can now govern the influential scope and moderating linkages [6]. Sarsted et al. [61] also proposed using Smart-PLS to produce a developmental model of observed and latent variables. Notably, a few pre-conditions must be met before the model can be validated. Smart-PLS software has been extensively utilized in various studies, including social and behavioral sciences and corporate management [7,52,53,84].

### 4.1 Descriptive statistics and correlations

Table 2 presents the model variables’ means, standard deviations, and inter-correlations. Interpersonal distrust associates positively with perceived workplace ostracism (r = 0.530, p > 0.01); perceived workplace ostracism associates positively with knowledge hiding (r = 0.667, p > 0.01); and person-organization unfit associates positively with perceived workplace ostracism and knowledge hiding (r = 0.378, p > 0.01) and (r = 0.395, p > 0.01), respectively.

**Table 2:** Model variables’ means, standard deviations, and inter-correlations.

Constructs	Mean	SD	1	2	3	4
1. Interpersonal distrust	2.60	.97	1.000			
2. Knowledge hiding	1.57	.81	0.272^a^	1.000		
3. Person-organization unfit	2.39	.88	0.372^a^	0.395^a^	1.000	
4. Perceived workplace ostracism	1.81	.76	0.530^a^	0.667^a^	0.378^a^	1.000

a p > 0.01 (2-tailed); SD = standard deviation.

### 4.2 Measurement model

The first data analysis phase evaluated the measurement model to verify its validity and reliability. Table 3 shows that all variables' indicator loadings are above the threshold of 0.60 [16]. All measures of reliability, including Cronbach's alpha, Dijkstra and Henseler's *rho*, and composite reliability, are greater than 0.70, demonstrating sufficient consistent reliability. Moreover, the measurement model has convergent validity because each variable's average variance extracted (AVE) is above 0.50 [32].

**Table 3:** Measurement model result.

Variables and Items	Indicator loadings	Cronbach's alpha	rho_A	Composite reliability (CR)	Average variance extracted (AVE)
Knowledge hiding		0.906	0.909	0.935	0.782
KH1	0.886				
KH2	0.828				
KH3	0.931				
KH4	0.888				
Interpersonal distrust		0.797	0.812	0.880	0.710
ID1	0.793				
ID2	0.875				
ID3	0.858				
Perceived workplace ostracism		0.948	0.950	0.956	0.684
PWO1	0.742				
PWO2	0.849				
PWO3	0.829				
PWO4	0.845				
PWO5	0.844				
PWO6	0.833				
PWO7	0.814				
PWO8	0.882				
PWO9	0.857				
PWO10	0.764				
Person-organization unfit		0.817	0.890	0.888	0.727
P–O1	0.738				
P–O2	0.909				
P–O3	0.899				

Lastly, discriminant validity, measuring the constructs’ uniqueness [72], was assessed using the heterotrait-monotrait ratio (HTMT), showing discriminant validity for all components as the values are below 0.85 [36] (see Table 4). The variance inflation factor (VIF) was used to test for collinearity. All VIF values are below the threshold of 5, indicating no collinearity concerns [32].

**Table 4:** Heterotrait-monotrait ratio (HTMT).

Variables	1	2	3	4
1. Interpersonal distrust	–			
2. Knowledge hiding	0.308	–		
3. Perceived workplace ostracism	0.604	0.717	–	
4. Person-organization unfit	0.451	0.435	0.409	–

### 4.3 Structural model

**Fig. 2:**
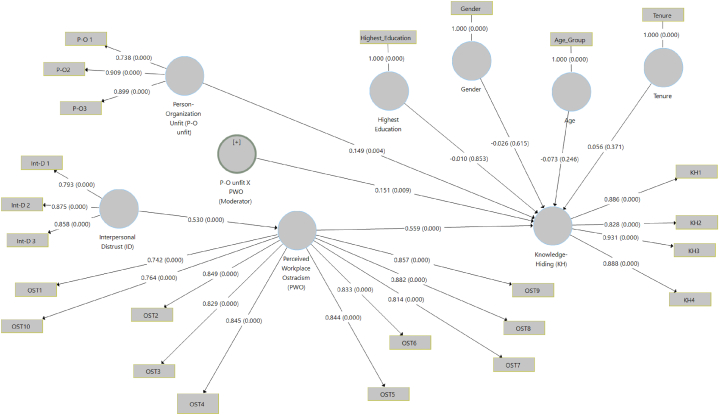
The structural model's results with path coefficients.

Path analysis was applied to verify the study's four hypotheses (see Table 5). First, we examined interpersonal distrust's impact on perceived workplace ostracism. Interpersonal distrust (β = 0.530, p < 0.01) positively relates to perceived workplace ostracism, supporting Hypothesis 1. Moreover, perceived workplace ostracism (β = 0.559, p < 0.01) positively relates to knowledge hiding, supporting Hypothesis 2. Furthermore, perceived workplace ostracism mediates the interpersonal distrust–knowledge hiding relationship (β = 0.294, p < 0.01), supporting Hypothesis 3. Finally, person-organization unfit moderates the perceived workplace ostracism–knowledge hiding relationship (β = 0.151, p < 0.01), supporting Hypothesis 4. Fig. 2 illustrates the results diagrammatically.

**Table 5:** Path analysis.

Hypotheses		Beta	t	P**	Decision
**H1**	Interpersonal distrust → Perceived workplace ostracism	0.530	10.3	0.000	Supported
**H2**	Perceived workplace ostracism → Knowledge hiding	0.559	10.8	0.000	Supported
**H3**	Interpersonal distrust → Perceived workplace ostracism → Knowledge hiding	0.294	7.7	0.000	Supported
**H4**	Perceived workplace ostracism * Person-organization unfit → Knowledge hiding	0.151	2.65	0.009	Supported

Remark: **p < 0.01.

The R^2^ value of the study's dependent and mediating variables [17] and the Stone-Geisser Q^2^ test for predictive relevance were analyzed to assess the structural model's quality [34]. The study's dependent variable (knowledge hiding) and mediating variable (perceived workplace ostracism) have R^2^ values of 0.47 and 0.28, respectively; all route coefficients are significant.

Lastly, Chin et al. [20] suggest using R^2^ and the Stone-Q^2^ Geisser's values to test the predictive value when assessing a PLS model. Thus, we performed SmartPLS blindfolding to assess the findings' consistency. The Q^2^ values are above zero for knowledge hiding (Q^2^ = 0.373) and perceived workplace ostracism (Q^2^ = 0.189), indicating that the model is stable and satisfies the requirement of predictive relevance.

The model fitness was analyzed using the standardized root mean squared residual (SRMR) [38]. The SRMR score of 0.053, i.e., below the threshold of 0.08 [38], indicates the model's good fit.

## 5 Discussion

In this research, we sought a holistic understanding of perceived workplace ostracism by examining its antecedents and consequences in a single study. It seems to be absent from the existing literature. In particular, we examined the impact of interpersonal distrust on perceived workplace ostracism and, subsequently, knowledge hiding. We further investigated the function of person-organization unfit as a moderator in the relationship between perceived workplace ostracism and knowledge hiding. Finally, we analyzed the effect of perceived workplace ostracism as a mediator between person-organization unfit and knowledge hiding. Based on our findings, all hypothesized relationships were supported (see Table 5).

We found that interpersonal distrust drives individuals to feel ostracized, as distrusting others hinders the process of identifying and associating with the social group, supporting hypothesis 1. This result aligns with past research [42,64,85] that shows a positive relationship between interpersonal distrust and perceived workplace ostracism. By building on social identity theory [70], we argue that employees who distrust colleagues are more likely to detach from the prototypical group and identify as outgroup members, fostering feelings of social exclusion.

Furthermore, our result demonstrates a significant correlation between perceived workplace ostracism and knowledge hiding, supporting Hypothesis 2. Previous research shows that perceived workplace ostracism significantly increases knowledge hiding behavior [58,86]. Based on the negative reciprocity principle [30], employees who perceive themselves as victims of workplace ostracism will view the perpetrator as causing harm to their interpersonal relationships. In response, they will feel obligated to act negatively by hiding relevant information when asked.

Moreover, our findings confirm the mediational effect of perceived workplace ostracism in the relationship between interpersonal distrust and knowledge hiding. Lack of trust makes employees feel excluded [42,64,85]. Using social identity theory [70], distrustful employees move away from the prototypical (trustful and cooperative) group, thereby identifying as outgroup members. As a result, employees who feel socially excluded reciprocate negatively by hurting others to restore their social functioning and system [18] by withholding knowledge (based on the negative reciprocity principle [30]), supporting Hypothesis 3.

Our findings further indicate that the mismatch between the employees and the organization's values (e.g., person-organization unfit) amplifies perceived workplace ostracism's influence on knowledge hiding, supporting Hypothesis 4. This finding offers a novel contribution to the current literature since none of the existing studies examined P–O unfit as a moderator in the relationship between PWO and its consequences. Misalignment between the employee's and organization's values (person-organization unfit) intensifies the employee's outgroup feelings and encourages knowledge-hiding behavior because it implies insufficient social identification with and belongingness to the organization. As knowledge hiding can offer a way to deviate from organizational norms and expectations [40], it can be seen as employees' attempts to restore social functions and systems [18].

## 6 Theoretical implications

By testing our theoretical model (see Fig. 1), our study contributes to the literature on perceived workplace ostracism in several ways. Firstly, our findings offer empirical insight into the significance of developing a holistic model of perceived workplace ostracism that incorporates antecedents and consequences in a single study. Much of the literature is quite fragmented, as past studies focused on either the antecedents or consequences of perceived workplace ostracism [37]. Our study bridges these two streams of research to offer a comprehensive picture of the antecedents and consequences of perceived workplace ostracism in a single study, which allows us to understand the nomological network between the constructs better. In our study, for instance, we can demonstrate that interpersonal distrust can lead to individuals feeling ostracized and excluded, as distrusting colleagues prevents them from identifying and associating with a social group, depriving them of meeting their belongingness needs [82]. Studies have shown the adverse effect of such unmet needs on organizational effectiveness [11]. On this note, our findings are consistent with the postulations of the social identity theory [70], where identifying oneself with a group allows one to develop self-esteem, self-control, and belongingness [74]. Furthermore, our result aligns with past research [42,64,85] as we found that employees who are distrustful toward their colleagues are more likely to detach themselves from the prototypical group and identify themselves as outgroup members, leading them to be socially excluded.

In addition to the social identity theory, our findings provide empirical support for the applicability of the negative reciprocity principle [30] in explaining the likelihood that perceived ostracized victims will engage in negative behavioral responses, such as hiding the requested knowledge. While we did not collect data on why individuals are distrustful toward their colleagues, we assume that past experiences that harm their interests can trigger interpersonal distrust [68]. Employees who perceive themselves as victims of workplace ostracism will view the perpetrator as causing harm to their interpersonal relationships. In response, they feel obligated to act negatively by hiding relevant information when asked. On this note, hiding knowledge can be interpreted to restore the balance and functions of social systems [18]. While this interpretation is reasonable, more studies are needed to validate it. More importantly, this finding raises an important question about the likely consequences of perceived workplace ostracism, which can jeopardize organizational functioning and well-being.

Finally, this study offers new knowledge by proposing and validating the intervening effect of person-organization unfit in the relationship between perceived workplace ostracism and knowledge hiding, as well as the mediating effect of perceived workplace ostracism in the relationship between interpersonal distrust and knowledge hiding.

## 7 Practical implications

Our findings offer several practical implications. First, as perceived workplace ostracism significantly impacts knowledge hiding, managers should strive to reduce it by fostering an honest and transparent environment. In addition, organizations should implement strategies towards goal-interdependence, task-interdependence, and group-based prize systems.

Second, we encourage firms to formulate job ads communicating their values and norms to ensure value congruence among applicants. Incorporating a pre-recruitment person-organization fitness exam would further ensure candidates’ alignment with the organizational culture.

Finally, fostering employees’ trust can help organizations address perceived workplace ostracism. It can be achieved through social gatherings, platforms, and programs, such as annual reunions, devoted to social interactions that foster social relationships and enhance employee trust.

## 8 Limitations and future research directions

This study acknowledges several potential limitations while offering exciting opportunities for future research. Firstly, our sample comprised individuals rooted in the UAE's collectivist culture, hindering the extrapolation of the findings to primarily individualistic cultures. Future studies should apply our model across different cultural contexts and industries to explore its universal applicability and replicability.

Secondly, we used convenience sampling, potentially limiting the findings' broader applicability. Future researchers are urged to consider probability sampling to strengthen the findings’ external validity and overall generalizability.

Third, the potential moderating role of transformational leadership in the perceived workplace ostracism–knowledge hiding relationship is intriguing. Prior research indicates that transformational leadership stimulates information sharing and discourages knowledge concealment [46]. Uncovering the nuances of this relationship would provide further insights for managing workplace dynamics.

These insights offer a solid foundation for future research. We urge researchers to elaborate upon our model, leveraging our findings to develop strategies addressing perceived workplace ostracism's devastating effects. It would yield tangible benefits, including healthier and more inclusive workplace environments.

## 9 Conclusion

These findings offer a fresh perspective on the complex picture of perceived workplace ostracism, its origins, and its repercussions. Our data lend credence to a paradigm where interpersonal distrust acts as a catalyst, amplifying workplace ostracism perceptions and subsequently triggering employees’ knowledge-hiding behavior. This comprehensive understanding is a valuable addition to the current knowledge on workplace ostracism, filling a gap ignored by all-inclusive models.

Furthermore, our research highlights a significant moderating influence of person-organization unfit within this dynamic. Specifically, the incongruity between the employee's and the organization's values intensifies perceived workplace ostracism's adverse effects on knowledge hiding.

Our UAE context adds distinctive cultural insights into the experience and impact of workplace ostracism. However, there is a need for future exploration to evaluate the findings’ applicability across varying cultural contexts and sectors.

Pragmatically, these findings equip organizations with implementable insights to foster healthier and more inclusive workspaces. By managing interpersonal distrust and encouraging greater alignment between individual and organizational values, companies can dampen the negative impacts of workplace ostracism and counter knowledge-hiding behavior, boosting overall organizational productivity and effectiveness.

This research calls for additional exploratory efforts in this domain, such as uncovering other moderating and mediating elements, thereby facilitating the development of more refined holistic models of workplace ostracism.

## Ethical compliance

This research was granted ethical approval from the Research Ethics Committee of the 10.13039/100016714University of Sharjah, ethics approval reference (REC-23-03-08-01-PG). The authors confirm that all participants gave informed consent to participate. The questionnaires were anonymized, and participants could withdraw from the study at anytime.

## Author contribution statement

Fatima Saeed Al-Dhuhouri: Conceived and designed the experiments; Performed the experiments; Analyzed and interpreted the data; Contributed reagents, materials, analysis tools or data; Wrote the paper.

Faridahwati Mohd Shamsudin: Conceived and designed the experiments; Wrote the paper.

## Data availability statement

The data that has been used is confidential.

## Declaration of competing interest

The authors declare that they have no known competing financial interests or personal relationships that could have appeared to influence the work reported in this paper.
